# Laryngeal Lymphoma in a Child – Case Report and Review of Literature 

**DOI:** 10.22038/IJORL.2022.57663.2984

**Published:** 2022-11

**Authors:** Anupriya Ayyaswamy, Prasanna Kumar Saravanam, Sneha Latha, Sandhya Sundaram

**Affiliations:** 1 Department of ENT, Head and Neck Surgery, Sri Ramachandra Institute of Higher Education and Research, Porur, Chennai; 3 Division of Pediatric Hemato Oncology, Sri Ramachandra Institute of Higher Education and Research, Porur, Chennai.; 4 Department of Pathology, Sri Ramachandra Institute of Higher Education and Research, Porur, Chennai.

**Keywords:** Child, Dysphonia, Lymphoma, Larynx, Snoring

## Abstract

**Introduction:**

Head and neck is the second most common region for lymphomas. Extranodal lymphomas of the larynx are rare in the pediatric population. Non Hodgkin Lymphoma (NHL) of the larynx is common in the supraglottic region as its rich in lymphoid tissue. They may present with dysphagia, dysphonia, snoring and progressive respiratory distress. Early visualization of the larynx is essential in such cases for appropriate diagnosis to improve the survival rates.

**Case Report:**

We present a case of 9 year old boy who presented with a change in voice, snoring and feeding difficulties for one year. Video laryngoscopy revealed globular mass arising from the epiglottis. He underwent excision biopsy and by immunohistochemistry was diagnosed to have diffuse large B cell lymphoma. He was treated with chemotherapy and the child is clinically well in the follow-up, 1 year after the completion of therapy.

**Conclusions:**

Although primary lymphomas of the larynx in children are rare, a high index of clinical suspicion is warranted to avoid diagnostic delays to initiate appropriate management to have better outcomes.

## Introduction

Primary extranodal lymphoma of the head and neck account for 2.5% of all lymphomas (1). Laryngeal lymphomas are very rare, accounting for 1 % of laryngeal malignancies and are mostly reported in the adult population (2). Only very few cases of epiglottis as the primary site of laryngeal lymphoma are reported in the pediatric age group. We report a case of 9 year old boy who was diagnosed with primary B cell lymphoma of the epiglottis, treated with surgery and chemotherapy and is currently well on follow-up. 

## Case Report

**Fig 1:**
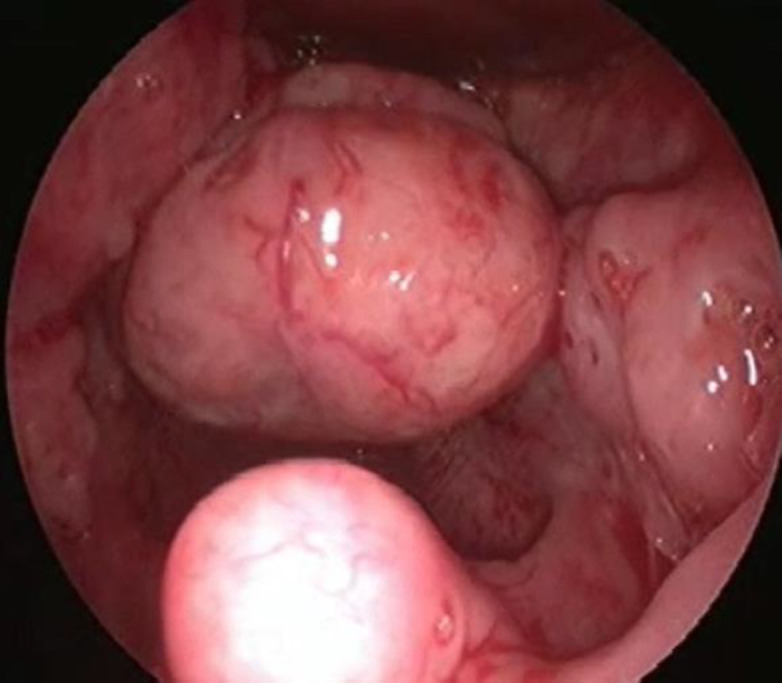
Video laryngoscopy showing a single globular mass with irregular surface arising from epiglottis extending till vallecula and base of tongue

A 9 year old boy presented with complaints of difficulty in swallowing, change in voice and snoring for one year associated with a weight loss of 4 kgs during this period. The child did not have any complaints of breathing difficulty, fever or night sweats. On examination the oral cavity was normal. There was no cervical lymphadenopathy. Indirect Laryngoscopy showed a single globular mass seen occupying the base of the tongue and epiglottis. Bilateral vocal cords could not be visualized. Video laryngoscopy done showed a single globular mass with irregular surface arising from epiglottis extending till vallecula and base of tongue (Figure 1). 

**Fig 2:**
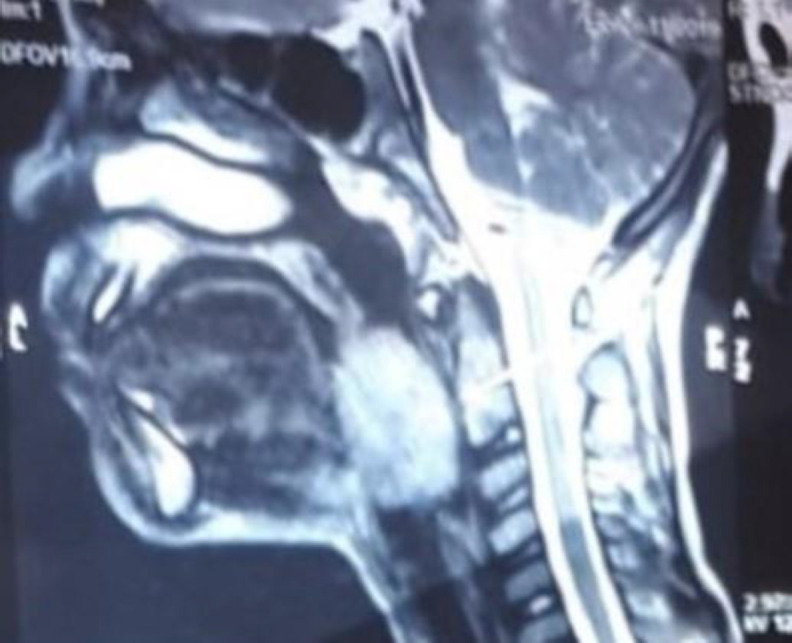
CT neck showing hyperdense lesion involving epiglottis extending into vallecula obstructing the airway

Fibre Optic Laryngoscopy showed bilateral vocal cords were mobile, pyriform fossa free on both sides. Contrast-enhanced computed tomography (CECT) neck was done to look for the extent of the lesion which showed hyperdense lesion measuring 2.8 x3.7 x3.5cms involving epiglottis extending into vallecula obstructing the airway (Figure 2).

**Fig 3:**
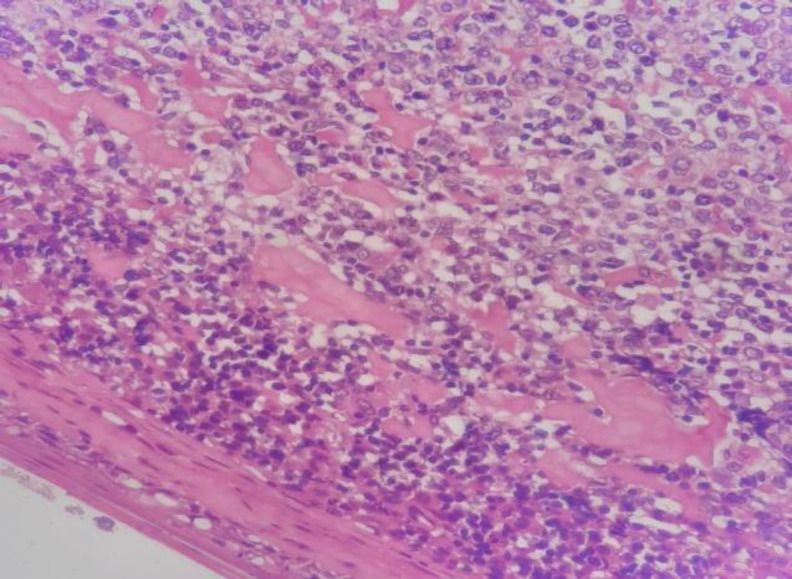
Histopathological image shows stratified squamous epithelium with underlying subepithelium showing sheets of a monomorphic population of lymphoid cells infiltrating into the mucosal and adjacent cartilaginous tissue

The patient underwent coblation assisted complete excision of the growth by ENT surgeons. Histopathological examination showed fragment tissues lined by stratified squamous epithelium with underlying subepithelium showing sheets of a monomorphic population of lymphoid cells infiltrating into the mucosal and adjacent cartilaginous tissue (Figure 3).

**Fig 4a and 4b:**
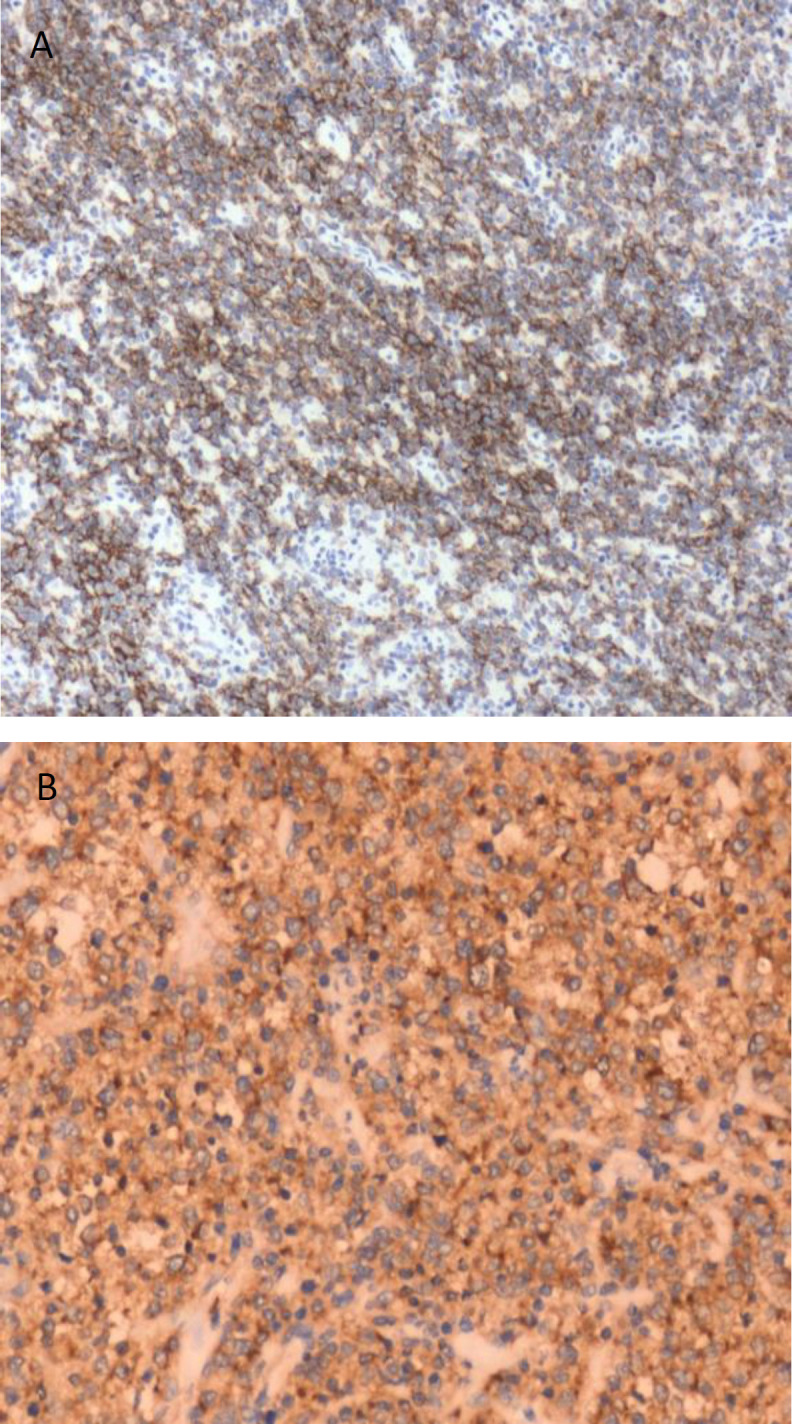
Immunohistochemistry positivity for CD 20 and CD 45

By immunohistochemistry, the cells were diffusely positive for CD20, CD45 (fig 4a and 4b) which was confirmative of B cell lymphoma. As part of the metastatic evaluation, whole-body PET CT and bone marrow biopsy done were normal. CSF analysis done to rule out CNS involvement was also normal. 

He was risk stratified as Group B disease and treated with chemotherapy as per LMP 96 protocol. He received COP (Cyclopho- sphamide, vincristine, prednisone), COPADM 1 and 2, (cyclophosphamide, vincristine, prednisone, doxorubicin, methotrexate) CYM1 & 2 (cytarabine, methotrexate) and intrathecal methotrexate and cytarabine. 

**Fig 5:**
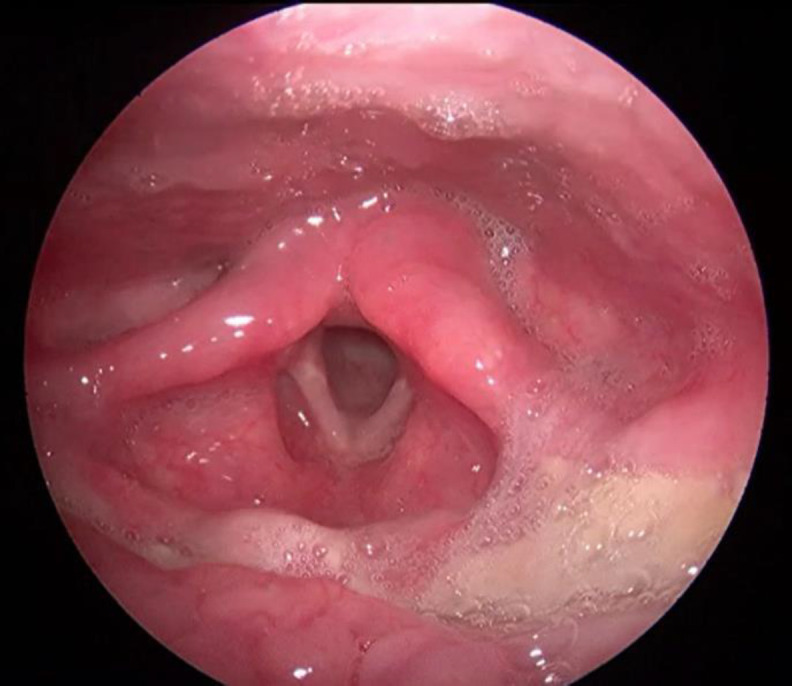
Video laryngoscopy done on follow up showing normal larynx

PET CT and video laryngoscopy done after completing chemo- therapy showed no evidence of recurrent or residual tumor. He was on regular follow up with pediatric oncology and ENT. A repeat video laryngoscopy done on follow up after 1 year of diagnosis showed normal findings (Figure 5). 

## Discussion

Head and neck is the second most common site for primary extranodal non-Hodgkin lymphoma, followed by gastrointestinal tract (1). NHL of the head and neck usually arises from the waldeyer’s ring. The other less commonly involved extranodal sites are sinonasal tract, salivary glands, thyroid and orbit. Laryngeal carcinomas account for <0.1% of all head and neck malignancies of childhood (3). Burkitt lymphoma, lymphoblastic lymphoma, diffuse large B cell lymphoma and anaplastic large cell lymphoma are the commoner NHL noted in children and adolescent age groups. Rhabdomyosarcoma and squamous cell carcinomas are the most common laryngeal tumors noted in pediatric patients (4). NHL of the larynx is very rare and B cell phenotype is more predominant accounting for more than 70% of cases (5). Among the NHL subtypes, large B cell Lymphoma and mucosa associated lymphoid tissue type marginal zone lymphomas are the most common primary laryngeal neoplasms (6). 

In laryngeal neoplasms, the most commonly involved site is the supraglottic region as its rich in follicular lymphoid tissue (7). A review of primary laryngeal lymphomas from 1996 -2008 cites an incidence of 47% in the supraglottic region, 25% in the glottic area and rest in subglottic or transglottic regions (8). 

Symptoms of laryngeal lymphoma include dysphonia, dysphagia, hoarseness of voice, cervical lymphadenopathy, cough, dyspnoea and occasionally systemic features like fever and weight loss might be present (Table 1) (9).

**Table 1:** The pediatric cases with laryngeal NHL is summarised below

**Study**	**Site of Lymphoma**	**Age/Gender**	**Clinical Features**	**Diagnosis**	**Management**	**Follow-Up**
Cohen et al 1987	Supraglottis Supra-glottis and Sub-glottis	4 years, 7 months/F 9 years 3 months/F	Croup / Laryngitis - 1 month Difficulty in breathing	Malignant small cell neoplasm Malignant NHL of Larynx	Chemotherapy and radiation Chemotherapy and radiation	2 years free of disease 15 years (2 years free of disease)
Palenzuela et al 2002	supraglottis, glottis, subglottis	15/M	progressive respiratory distress, profuse night sweats	EBV related B cell lymphoma	chemotherapy radiotherapy, tumor debulking by laser	died during treatment due to lung infection
Rodriguez H et al 2014	Epiglottic invasion and Glottic narrowing	8 years/ M	Dysphony and progressive respiratory failure x 3 years	Lymphoblastic T cell Lymphoma	Chemotherapy	Child died 16 months after diagnosis due to Septic Shock during 2^nd^ line chemotherapy
Amanda Martin et al 2017	Supraglottis	14/F	Dysphagia x few monthsWeight loss – 14lbs	Mature B cell Lymphoma	Surgery and chemotherapy	1 month free of disease
Paloma et al 2019	Right pyriform fossa	13/M	Rapidly enlarging right neck mass	Diffuse large B Cell Lymphoma	Surgery and chemotherapy	Not available

Laryngeal lymphomas remain localized for a longer duration without progression of symptoms, and a low index of suspicion in children leads to diagnostic delays. The symptoms are generally attributed to prepubertal voice changes or respiratory infections. For a child with laryngeal mass, the differential diagnosis to be considered are cystic lesions, recurrent respiratory papillomatosis, and laryngeal malignancies. In the presence of a submucosal mass centered in the supraglottis in imaging studies, NHL should be considered if there is a superior extension to the oropharynx or nasopharynx (10). 

The biopsy of the lesion is essential to determine the pathology and guide treatment options. Chemotherapy is the treatment of choice. Diffuse Large B Cell Lymphoma is treated like Burkitt lymphoma. Surgery is recommended in cases of extensive airway compromise (11). Though children have more aggressive forms of non-Hodgkins lymphoma, with adequate and appropriate treatment five-year survival rate is high up to 5 years in 90% of children (12). 

## Conclusion

Though laryngeal neoplasms are rare in children, symptoms like voice change, dysphagia, and dyspnoea need extensive evaluation with a direct laryngoscope to rule out laryngeal masses. As the treatment outcomes are better with chemotherapy, early diagnosis is essential in such cases.
